
JOURNAL INFORMATION
==============================
NLM Title Abbreviation: ScientificWorldJournal
Journal ID: ScientificWorldJournal
NLM Journal ID: 101131163
Journal ID: TSWJ

The Scientific World Journal

ISSN: 2356-6140
EISSN: 1537-744X
Publisher: Wiley

ARTICLE INFORMATION
==============================
PMCID: PMC5901262
PMID: 17982575
DOI: 10.1100/tsw.2007.215
Article ID: 725103
Article version: 1
Subjects: Editorial

Heteromerization of G-Protein–Coupled Receptors. Implications for Central Nervous System Function and Dysfunction

Ferré Sergi *
National Institute on Drug Abuse, Intramural Research Program, National Institutes of Health, Department of Health and Human Services, Baltimore, MD 21224, USA
*Sergi Ferré: sferre@intra.nida.nih.govAcademic Editors: D. Shurtleff and S. Ferre

Publication date: 2007
Electronic publication date: 2007 Nov 2
Volume: 7
First page: 46
Last page: 47
Received 2007 Jul 25; Accepted 2007 Jul 25
Copyright: Copyright © 2007 Sergi Ferré
Copyright year: 2007
License: This is an open access article distributed under the Creative Commons Attribution License, which permits unrestricted use, distribution, and reproduction in any medium, provided the original work is properly cited.
License URL: https://creativecommons.org/licenses/by/3.0/

==============================
Editorial
NIDA Special Issue on Frontiers in Addiction Research
*TheScientificWorldJOURNAL* (2007) 7(S2), 46–47
ISSN 1537-744X; DOI 10.1100/tsw.2007.215

TheScientificWorldJOURNAL
www.thescientificworld.com

### Heteromerization of G-Protein–Coupled Receptors. Implications for Central Nervous System Function and Dysfunction

Sergi Ferré

*National Institute on Drug Abuse, Intramural Research Program, National Institutes of Health, Department of Health and Human Services, Baltimore, MD 21224*

**E-mail:** sferre@intra.nida.nih.gov

*Received July 25, 2007; Accepted July 25, 2007; Published November 2, 2007*

---

Like many new concepts, especially those that break old dogmas, it took some time for the scientific community to accept the existence and functional significance of G-protein–coupled receptor (GPCR) heteromerization (for a recent review, see Ferré et al.[1]). As reviewed in four manuscripts of this "NIDA Special Issue", it is becoming clear that GPCR heteromerization plays a very important role in central nervous system (CNS) function and that GPCR heteromers can become invaluable targets for drug therapy.

Very often when an important new concept is generally accepted, numerous claims of its paternity appear, often by scientists that initially were completely against its acceptance. In the present issue, Franco et al.[2] provide an historical account of the discovery of receptor heteromers. It was at the beginning of the 1990s that Agnati, Fuxe, and coworkers hypothesized that their initially discovered "intramembrane receptor-receptor interactions" (reviewed in Agnati et al.[3]) required a close intermolecular cross-talk, which implied receptor heteromerization[4]. Fluorescence and Bioluminescence Resonance Energy Transfer (FRET and BRET) techniques allowed the demonstration of receptor heteromers in transfected cells in culture[5], but these techniques could not be applied *in situ* in natural tissues. Therefore, a major challenge has been the identification of receptor heteromers in the brain. Franco et al. review the concept of "biochemical fingerprint", the most useful practical method to detect receptor heteromers in the brain[2]. Basically, this method involves the identification of a biochemical characteristic of the receptor heteromer in the brain after its initial demonstration in an artificial cell system. In fact, intramembrane receptor-receptor interactions constitute a common biochemical fingerprint of receptor heteromers[1,2,3].

The biochemical fingerprint of the dopamine $D_1$-$D_2$ receptor heteromer, reviewed by George and O'Dowd[6], is its characteristic G-protein coupling ($G_{q/11}$), which leads to a specific calcium-dependent PLC signaling pathway. The discovery of the $D_1$-$D_2$ receptor heteromer provided an explanation for previous experimental findings that show the ability of $D_1$ receptors to couple to $G_{q/11}$ in native tissue, but not in transfected cells expressing $D_1$, but not $D_2$ receptors[7]. An additional discovery about the $D_1$-$D_2$ receptor heteromer is that it can be selectively activated by specific ligands[7]. Conceptually, this illustrates a very important property of receptor heteromers: They can be targeted by specific drugs. Therefore, identification of receptor heteromers that modulate brain functional units particularly involved in brain dysfunction becomes an important goal in the field.

Heteromerization of mu and delta opioid receptors (μO and δO receptors, respectively) also promote the formation of novel signaling, as reviewed by Rozenfeld et al.[8]. Activation of μO receptors leads to a G-protein–mediated rapid and transient MAPK activation, while activation of μO-δO receptor heteromers

©2007 with author.
Published by TheScientificWorld; www.thescientificworld.com leads to a switch to a G-protein–independent and β-arrestin–mediated delayed, but sustained, MAPK activation[9]. A number of studies indicate that morphine tolerance correlates with formation of μO-δO receptor heteromers, and Rozenfeld et al. suggest that a switch to the β-arrestin–mediated signaling contributes to the changes in morphine response that underlie tolerance[8]. In fact, targeting the μO-δO receptor heteromer with morphine, which activates μO receptor, plus a δO receptor antagonist, diminishes morphine tolerance and enhances analgesia[8].

As reviewed by Ferré et al.[10], the various localizations (synaptic, extrasynaptic), sources (neurons, glial cells), and modes of neurotransmission (synaptic and extrasynaptic) impinging on the receptor heteromers allow them to play a key role in the processing of computations performed by local modules, defined as the minimal portion of one or more neurons and/or one or more glial cells that operates as an independent integrative unit[11]. Ferré et al. provide an example of the integrative role of adenosine receptor heteromers as processors of information in the striatal spine module[10]. Targeting those adenosine receptor heteromers might provide new treatments for basal ganglia disorders and drug addiction. In fact, targeting the adenosine $A_{2A}$-$D_2$ receptor heteromer with a dopamine agonist (L-dopa) plus an $A_{2A}$ receptor antagonist is being shown to be successful in clinical trials in Parkinson’s disease[12].

### REFERENCES

1. Ferré, S., Ciruela, F., Woods, A.S., Lluis, C., and Franco, R. (2007) Functional relevance of neurotransmitter receptor heteromers in the central nervous system. *Trends Neurosci.,* in press.
2. Franco, R., Casadó, V., Cortés, A., Ferrada, C., Mallol, J., Woods, A., Lluis, C., Canela, E.I., and Ferré, S. (2007) Basic concepts in G-protein-coupled receptor homo- and heterodimerization. *TheScientificWorldJOURNAL* **7(S2)**, 48-57.
3. Agnati, L.F., Ferré, S., Lluis, C., Franco, R., and Fuxe, K. (2003) Molecular mechanisms and therapeutical implications of intramembrane receptor/receptor interactions among heptahelical receptors with examples from the striatopallidal GABA neurons. *Pharmacol. Rev.* **55,** 509–550.
4. Zoli, M., Agnati, L.F., Hedlund, P.B., Li, X.M., Ferré, S., and Fuxe, K. (1993) Receptor-receptor interactions as an integrative mechanism in nerve cells. *Mol. Neurobiol.* **7,** 293–334.
5. Bouvier, M. (2001) Oligomerization of G-protein-coupled transmitter receptors. *Nat. Rev. Neurosci.* **2,** 274–286.
6. George, S.R., and O’Dowd, B.F. (2007) A novel dopamine receptor signaling unit in brain: Heterooligomers of D1 and D2 dopamine receptors. *TheScientificWorldJOURNAL* **7(S2)**, 58-63.
7. Rashid, A.J., So, C.H., Kong, M.M., Furtak, T., El-Ghundi, M., Cheng, R., O'Dowd, B.F., and George, S.R. (2007) D1-D2 dopamine receptor heterooligomers with unique pharmacology are coupled to rapid activation of Gq/11 in the striatum. *Proc. Natl. Acad. Sci. U. S. A.* **104,** 654–659.
8. Rozenfeld, R., Abul-Husn, N.S., Gomes, I., and Devi, L.A. (2007) Emerging roles of delta opioid receptors in the regulation of mu opioid receptor function. *TheScientificWorldJOURNAL* **7(S2)**, 64-73.
9. Rozenfeld, R. and Devi, L.A. (2007) Receptor heterodimerization leads to a switch in signaling: β-arrestin2-mediated ERK activation by μ-δ opioid receptor heterodimers. *FASEB J.,* in press.
10. Ferré, S., Ciruela, F., Quiroz, C., Luján, R., Popoli, P., Cunha, R.A., Agnati, L.F., Fuxe, K., Woods, A.S., Lluis, C., and Franco, R. (2007) Adenosine receptor heteromers and their integrative role in striatal function. *TheScientificWorldJOURNAL* **7(S2)**, 74-85.
11. Ferré, S., Agnati, L.F., Ciruela, F., Lluis, C., Woods, A.S., Fuxe, K., and Franco, R. (2007) Neurotransmitter receptor heteromers and their integrative role in 'local modules': The striatal spine module. *Brain Res. Rev.* Epub ahead of print.
12. Jenner, P. (2003) Istradefylline, a novel adenosine A2A receptor antagonist, for the treatment of Parkinson's disease. *Expert Opin. Investig. Drugs* **14,** 729–738.

**This article should be cited as follows:**

Ferré, S. (2007) Heteromerization of G-protein–coupled receptors. Implications for central nervous system function and dysfunction. *TheScientificWorldJOURNAL* **7(S2)**, 46–47. DOI 10.1100/tsw.2007.215.
